# The effect of intravenous and inhalation anesthesia in general on the cognition of elderly patients undergoing non-cardiac surgery: a systematic review and meta-analysis

**DOI:** 10.3389/fmed.2023.1280013

**Published:** 2023-11-15

**Authors:** Leilei Huang, Yong Zhang

**Affiliations:** Department of Anesthesiology, The First Affiliated Hospital of Guangzhou University of Chinese Medicine, Guangzhou, Guangdong, China

**Keywords:** intravenous anesthesia, inhalation anesthesia, non-cardiac surgery, the elderly, cognitive function

## Abstract

**Background:**

Postoperative cognitive dysfunction (POCD) is a postoperative complication that often occurs in the elderly. This systematic review and meta-analysis aimed to compare intravenous anesthetics (propofol) with inhalation anesthetics (sevoflurane) regarding the occurrence of POCD in the elderly who underwent non-cardiac surgery.

**Methods:**

The investigators searched for published articles from the PubMed, Embase, Web of Science, Scopus, Cochrane, and Clinicalkey databases. Clinical studies comparing the incidence of POCD in elderly patients undergoing intravenous or inhalation anesthesia in general were selected. Primary outcomes included the occurrence of POCD at 1, 3, and 7 days. The secondary outcomes were the patient’s plasma S-100β protein levels (pg*mL^−1^) and delayed neurocognitive recovery incidence 5–7 days after surgery.

**Results:**

Fifteen studies including 3,817 patients were enrolled in the systematic review. Ten studies involving 1,829 patients were enrolled in the meta-analysis. The results demonstrate that there was no difference between the intravenous and inhalation groups in the incidence of POCD within 1–7 days (95% CI 0.73–1.26, *p* = 0.77) and the occurrence of delayed neurocognitive recovery 5–7 days after surgery (95% CI −353.15 to −295.44, *p* = 0.28). Plasma S-100β protein levels in the intravenous anesthesia group were lower than those in the inhalation group (95% CI 0.48–1.24, *p* < 0.001).

**Conclusion:**

For elderly patients undergoing non-cardiac surgery, inhalation anesthesia was comparable to intravenous anesthesia in terms of the occurrence of short-term POCD. Inhalation anesthesia may cause greater damage to the nervous system, with delayed recovery of cognitive function after 5–7 days showing no difference.

**Systematic review:**

identifier (CRD42021251317).

## Introduction

Among patients undergoing surgery, the proportion of older people is gradually increasing. Compared with adults, elderly patients are more likely to have postoperative cognitive dysfunction (POCD). Age is a risk factor for patients with (POCD) (1–3). The study by Kotekar et al. (4) found that the incidence of POCD was significantly greater in the 71–80-year-old group than in the 61–70-year-old group, while the rate of POCD in the 80-year-old group could reach 100%. POCD can severely impact the length of hospital stay and increase morbidity and mortality, especially in elderly patients undergoing surgery under general anesthesia (1, 2, 5–7). In non-cardiac surgery, older patients are at high risk for POCD. In non-cardiac surgery, 25–56% of older patients are affected by POCD in the first week after surgery (8).

The mechanism of POCD is currently unknown, and the effects of anesthesia on the occurrence of POCD during general anesthesia have been progressively realized (9). Sevoflurane, as a commonly used inhaled anesthetic, is believed to increase the incidence of POCD in older patients (10). Most recent animal experiments have focused on the effects of inhaled anesthetics on neurological function (11, 12), and the results of these seem to be unfavorable for the use of inhaled anesthetics in elderly patients (13). However, results from a number of studies have shown that sevoflurane intervention does not impair learning and memory. In some studies (14–16), it has been pointed out that intravenous and inhaled anesthetics have a neuroprotective effect in brain injury.

Currently, clinical studies on the impact of inhaled and intravenous anesthetics on the occurrence of POCD in older adults are relatively common, but a multi-center randomized controlled trial (RCT) (3) published in 2021 further investigated this controversial issue. After our search, however, there is no meta-analysis of studies published after 2018 on this topic. Therefore, we believe that a new study of this issue, in conjunction with recent clinical studies, is warranted. The purpose of this study is to conduct a systematic evaluation and meta-analysis to compare the effects of intravenous anesthetics (propofol) and inhalation anesthetics (sevoflurane) on the occurrence of POCD due to non-cardiac surgery in the elderly.

## Methods

### Study selection

Our research has been registered with PROSPERO under registration number CRD42021251317. The systematic review and meta-analysis were performed according to Preferred Reporting Items for Systematic Reviews and Meta-analyses (PRISMA) guidelines. We followed the PRISMA checklist to complete the meta-analysis. The researchers searched for articles published before 18 April in the PubMed, Embase, Web of Science, Scopus, Cochrane, and Clinicalkey databases. The search terms were: (cognition OR cognitive disorder OR cognitive deficit OR cognitive impairment OR cognitive function impairment OR cognitive dysfunction) AND (elderly patients OR aged OR the aged OR old people OR the elderly OR elder OR agedness) AND (volatile anesthetic OR inhalation anesthetic OR sevoflurane OR inhaled anesthetics OR inhalational anesthetic OR intravenous anesthetic OR TIVA OR total intravenous anesthesia OR propofol OR general anesthesia). The restrictive conditions for all search formulae were to search for titles, abstracts, and keywords. We had no restrictions on language. The searched literature was managed with EndNote X9 (Thomson Reuters, NY, United States). After excluding duplicates and non-clinical studies, the titles and abstracts were screened by the researcher. Finally, the researchers determined the included literature based on the full text. In addition, POCD did not include postoperative delirium in this study.

### Eligibility criteria

The studies included in the meta-analysis must meet the following criteria: clinical studies, comparison of intravenous anesthesia (propofol) and inhalation anesthesia in general (sevoflurane), and elderly patients receiving noncardiac surgery. Studies with the following characteristics were excluded: animal studies, study protocol, reviews, guidelines, conference abstract, without control, and different from inclusion criteria (not the elderly, different interventions, etc.).

### Risk of bias assessment

The investigators used the Cochrane collaboration tool to obtain the overall bias of the included studies and used RevMan 5.3 (Review Manager. Version 5.3. Copenhagen: The Nordic Cochrane Centre, The Cochrane Collaboration, 2014.) to make a risk of bias graph and summary.

### Data extraction

We extracted the characteristics of the included studies, including source, location of study, year of publication, design, eligible population, operations, study period, and number of patients. The patient and intervention characteristics of the studies were evaluated in the meta-analysis. The data for the meta-analysis were extracted by L.L.H. and checked with Y.Z.

### Outcomes

The primary outcome of this meta-analysis was the incidence of postoperative cognitive dysfunction (POCD) at 1, 3, and 7 days. The secondary outcomes were the patient’s plasma S-100β protein levels (pg*ml^−1^) and delayed neurocognitive recovery incidence 5–7 days after surgery.

### Statistical analysis

RevMan 5.3 was used for all data analysis in this study. The inverse variance random effects model was used to analyze continuous variables and expressed as the mean difference (MD) of the 95% confidence interval (CI). For binary variables, we reported the odds ratios (OR) and used the Mantel–Haenszel method for analysis. For the data with *p* < 0.05 or I (2)>50% for heterogeneity detection, the random-effect model was used for analysis, while data with *p* > 0.05 or I (2)≤50% were selected for the fixed-effect model. If only one study is included, the fixed-effect model is also selected. For the results with high heterogeneity [I (2)≤75%], we conducted a sensitivity analysis to exclude studies with high heterogeneity. Moreover, we performed a subgroup analysis to assess the incidence of POCD in different time periods. The intravenous group was the reference group for OR calculations.

## Results

### Literature search findings

**Figure 1:**
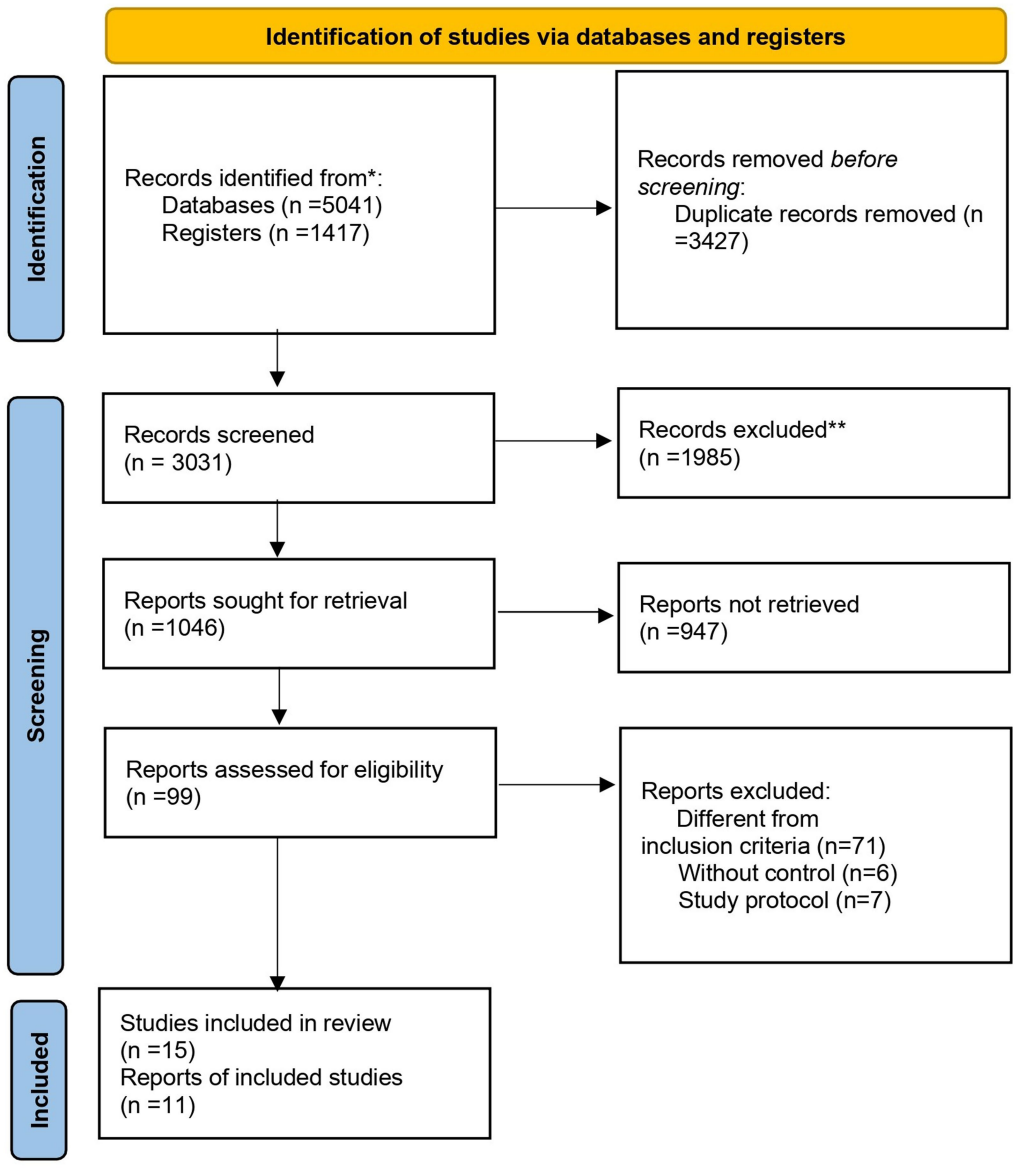
PRISMA flow diagram of search strategy and included studies.

By searching the PubMed, Embase, Web of Science, Scopus, Cochrane, and Clinicalkey databases for literature titles, abstracts, and keywords, 6,458 articles were obtained. We used EndNote X9 to find duplicates, leaving 5,412 articles and preserving 947 clinical trials. The abstracts and titles of the remaining studies were screened, and 99 were related to intravenous anesthesia, inhaled anesthesia, or elderly patients. Through the screening of the full text of these studies, 15 included studies were finally determined (Study protocol: 7; Without control: 6; Different inclusion criteria: 70). The literature retrieval process is illustrated in Figure 1. Of the 15 included studies, we screened 11 for meta-analysis that included both primary and secondary outcomes.

### Study and patient characteristics

We summarize the included studies’ characteristics in Table 1, including a total of 15 studies and 3,817 patients. Among these, 3,652 patients in 12 studies were from China [Shanghai (17–19), Jiaxing (5), Harbin (20), Guangxi (21), Guangdong (3), Jiangxi (22), Shandong (10), Chengdu (23), Beijing (24), and Shenzhen (25)], and the other three were from the USA (Kentucky) (26), Japan (Sapporo) (27), and Greece (28). Moreover, there were 13 studies published after 2015. Among the included studies, only one (22) was a retrospective study, and the rest were RCTs. Among the RCTs, four studies (10, 20, 21, 23) were double-blind and one was a multi-center study (3). All patients underwent non-cardiac surgery.

**Table 1:** Summary of the characteristics of the included studies (*n* = 14).

Source	Location of study	Year of publication	Design	Eligible population	Operations	Study period	Number of patients
Chen et al.	Jiaxing, China	2018	RCT	Elderly patients who met ASA I and II criteria	Elective moderate orthopedic surgery	NR	200
Geng et al.	Harbin, China	2017	RCT (double-blind)	Patients with ASA II–III, age ≥ 65 years, and a sufficient level of education	Laparoscopic cholecystectomy	December 2010 to June 2011	150
Guo et al.	Guangxi, China	2020	RCT (double-blind)	Age ≥ 65 years; ASA I, II, or III; elective tumor resection under general anesthesia; fluent in Chinese (speaking and reading); able to independently complete the neuropsychological tests.	Elective tumor resection	1 December 2016 to 31 December 2017	234
Li et al.	Guangdong, China	2021	Multicenter, RCT	Patient’s age was 60 years or older; surgery was expected to last 2 h or longer; patients did not have serious hearing and vision impairment and were able to read.	Elective major laparoscopic abdominal surgery	23 March 2013 to 11 March 2019.	544
Liang et al.	Jiangxi, China	2018	Retrospectively study	Patients with abdominal operation after definite diagnosis, age ≥ 60 years, had complete medical records, and were not treated in other hospitals.	Acute appendicitis operation. Cholecystitis operation. Intestinal obstruction operation. Gastrointestinal tumor operation.	January 2015 to December 2017	371
Liu et al.	Shanghai, China	2017	RCT	112 elderly patients treated with laparoscopic colorectal resection at Seventh People’s Hospital of Shanghai University of TCM.	Laparoscopic colorectal resection	January 2015 to January 2016	112
Mei et al.	Shanghai, China	2020	RCT	Age ≥ 60 years. Scheduled for surgery under general anesthesia. ASA I to III; MMSE score of more than 24 of 30. Chinese Mandarin as the native language. Having verbal communication capability and writing skills and thus being able to provide informed consent.	Hip/knee replacements	June 2016 to November 2019	209
Micha et al.	Greece	2016	RCT	Aged 60–74, scheduled for a noncardiac operation of more than 2 h duration	Noncardiac operation	June 2010 to July 2013	80
Nishikawa et al.	Sapporo, Japan	2004	RCT	ASA I or II; age ≥ 65 years; scheduled for elective laparoscope-assisted surgical procedures which would last more than 3 h under combined general and epidural anesthesia	Choledocholithotomy. Colectomy. Sigmoidectomy.	NR	55
Qiao et al.	Shandong, China	2015	RCT (double-blind)200	ASA I, II, or III; a sufficient level of education to be capable of completing neuro-psychological tests; preoperative MMSE score ≥ 23; no evidence of cardio-vascular, respiratory, or central nervous system disease; normal renal and hepatic function; no serious hearing or visual impairment; absence of a history of benzodiazepine or antidepressant use, alcohol or cigarette misuse or drug dependence; and no contraindication to propofol or inhalational anesthesia.	Esophageal carcinoma resection	January 2013 to December 2014	90
Rohan et al.	Kentucky, USA	2005	RCT	After hospital Ethics Committee approval and written, informed consent, age > 65 years presenting for minor urological or gynecological surgery, requiring general anesthesia, and with an anticipated hospital stay of one night postoperatively	Rigid cystoscopy. Transurethral resection of bladder mucosal tumor. Hysteroscopy	NR	30
Tang et al.	Chengdu, China	2014	RCT (double-blind)	Elderly patients with MCI. age > 60 years. ASA I–III.	Radical rectal resection surgery (Miles type)	January 2010 to November 2013	220
Yu	Shanghai, China	2017	RCT	Age > 60 years; having clear logical thought and verbal expression ability as well as the normal thinking before surgery; exclusion of cardiovascular and cerebrovascular diseases, incomplete renal function injury, central nervous system disease and mental disease; not receiving any drug therapy influencing the nervous system.	General thoracic surgery.	March 2014 to March 2016	1,000
Zhang et al.	Beijing, China	2018	RCT	Age > 65 and < 90 years; primary cancer without any radio or chemotherapy before surgery; scheduled to undergo surgery for cancer with an expected duration≥2 h under general anesthesia.	Major cancer surgery (≥2 h)	1 April 2015 to 15 October 2016	392
Ding et al.	Shenzhen, China	2021	RCT	(1) Age ≥ 65 years old; (2) no history of immune system disease; (3) no obvious abnormal liver and kidney function was found on blood biochemical examination; (4) normal coagulation; (5) no infection before surgery; (6) no anti-inflammatory or anticoagulant drugs were used within 2 weeks before surgery.	Underwent abdominal surgery	Aug 2019 to Aug 2020	130

MMSE, Mini-Mental State Examination; ASA, American Society of Anesthesiologists; RCT, Randomized Controlled Trial; NR, No record.

In the meta-analysis stage, we excluded five studies lacking outcome indicators and only evaluated 1,829 patients in 10 studies (all RCTs). Table 2 shows the characteristics of the patients in the evaluated studies. Only the study by Geng et al. (20) did not report the age of the patients, and the mean or median age of all other patients was >64 years. Six studies reported on BMI and there were no obese patients (BMI ≥ 30 kg/m^−2^). Only in three studies did the number of female patients exceed the number of male patients. The studies by Geng et al. (20) and Guo et al. (21) did not include ASA I patients, while the study by Nishikawa et al. did not include ASA III patients. In the study by Rohan et al. (26), the duration of anesthesia was the shortest, and in Zhang et al. (24) it was the longest. Qiao et al. (10) did not report it.

**Table 2:** Patient characteristics in the evaluated studies (*n* = 10).

Source, Yr	n	Intervention	Duration of anesthesia (h)	Age (years)	BMI (kg/m^−2^)	Male/Female	ASA class (I/II/III)
Geng et al., 2017	50	Propofol	Median (IQR): 1.21 (1.07, 1.81)	NR	Mean (SD) 24.37 (2.34)	20/30	0/35/15
	50	Sevoflurane	Median (IQR): 1.47 (1.17, 1.88)	NR	Mean (SD) 24.06 (2.23)	22/28	0/31/19
Guo et al., 2020	117	Propofol	Median (IQR): 4.45 (3.725, 5.142)	Median (IQR): 69.0 (66.0, 72.5)	Median (IQR): 22.8 (22.1, 23.5)	71/46	0/101/16
	117	Sevoflurane	Median (IQR): 4.37 (3.775, 5.317)	Median (IQR): 69.0 (66.0, 74.0)	Median (IQR): 22.9 (22.4, 23.6)	76/41	0/99/18
Li et al., 2021	226	Propofol	Median (IQR): 4.0 (3.3, 4.7)	Median (IQR): 64 (62, 68)	Median (IQR): 22.5 (20.2, 24.3)	169/57	18/178/30
	221	Sevoflurane	Median (IQR): 4.3 (3.4, 5.2)	Median (IQR): 65 (62, 69)	Median (IQR): 22.3 (20.3, 24.7)	145/76	21/170/30
Liu et al., 2017	56	Propofol	Mean (SD) 2.76 (0.18)	Mean (SD) 74.16 (4.21)	NR	31/25	NR
	56	Sevoflurane	Mean (SD) 2.67 (0.29)	Mean (SD) 75.82 (4.17)	NR	31/25	NR
Mei et al., 2020	106	Propofol	Mean (SD) 2.14 (0.56)	Mean (SD) 70.9 (6.7)	Mean (SD) 25.4 (3.7)	34/72	4/90/12
	103	Sevoflurane	Mean (SD) 2.21 (0.73)	Mean (SD) 71.5 (6.8)	Mean (SD) 26.1 (3.5)	27/76	5/78/20
Nishikawa et al., 2004	25	Propofol	Mean (SD) 4.75 (1.05)	Mean (SD) 71 (8)	NR	13/12	7/18/0
	25	Sevoflurane	Mean (SD) 4.33 (1)	Mean (SD) 71 (7)	NR	12/13	6/19/0
Qiao et al., 2015	30	Propofol	NR	Mean (SD) 68 (2)	Mean (SD) 24.41 (1.52)	21/9	NR
	30	Sevoflurane	NR	Mean (SD) 68 (3)	Mean (SD) 23.65 (1.14)	22/8	NR
Rohan et al., 2005	15	Propofol	Median (IQR): 0.3 (0.13, 0.5)	Median (IQR): 72.9 (65, 83)	NR	12/3	NR
	15	Sevoflurane	Median (IQR): 0.25 (0.17, 0.47)	Median (IQR): 73.8 (67, 86)	NR	11/4	NR
Tang et al., 2014	101	Propofol	Mean (SD) 2.58 (0.23)	Mean (SD) 69.6 (4.8)	NR	26/75	NR
	99	Sevoflurane	Mean (SD) 2.60 (0.24)	Mean (SD) 70.0 (4.3)	NR	32/67	NR
Zhang et al., 2018	195	Propofol	Median (IQR): 4.93 (3.82, 5.78)	Mean (SD) 72.8 (5.5)	Mean (SD) 23.6 (3.1)	135/60	12/146/37
	192	Sevoflurane	Median (IQR): 4.57 (3.62, 5.4)	Mean (SD) 72.4 (5.6)	Mean (SD) 24.0 (3.1)	128/64	20/142/30

SD, standard deviation; NR, no records; IQR, interquartile range; Yr, year; BMI, Body Mass Index.

### Intervention characteristics

We summarize the intervention characteristics of the evaluated studies in Table 3. Of the 10 studies evaluated, four selected different narcotic induction methods in groups P and S, respectively. In the study by Liu et al. (17), 1.5 mg/kg propofol +2 μg/kg remifentanil +0.1 mg/kg vecuronium bromide intravenous was chosen in group P and 2 mg/kg propofol +2 μg/kg remifentanil +0.1 mg/kg vecuronium bromide in group S. In the study of Nishikawa et al. (27), a targeted propofol concentration of 4 mg/mL using a computer-assisted TCI system was chosen in group P and 5% sevoflurane and 100% oxygen at 6 L / min until the inspired-limb drug concentration was >4% in group S. Rohan et al. (26) used target concentrations of propofol that were adjusted to maintain adequate depth of anesthesia in group P and the incremental dose, tidal volume inhalation induction technique in group S. Tang et al. (23) used a standard induction protocol: midazolam (0.03–0.04 mg/kg, i.v.), fentanyl (0.002–0.003 mg/kg, i.v.), and vecuronium (0.15–0.2 mg/kg, i.v.), and chose propofol (1.5–2.0 mg/kg, i.v.) in group P and 8% sevoflurane (FGF 6 L/min, inhalation, decreased after loss of consciousness to 3–4%, FGF 1–2 L/min) in group S.

**Table 3:** Intervention characteristics in the evaluated studies (*n* = 10).

Source, Yr	Anesthesia induction	n	Intervention	Anesthesia maintenance	Adjunct
Geng et al., 2017	Midazolam (0.05 mg·kg^−1^), fentanyl (4 μg·kg^−1^) and rocuronium (0.6 mg·kg^−1^). All patients received a TCI of 3.0 μg·mL^−1^ propofol	50	Propofol	Propofol (target concentration 2.5–3.0 μg·mL^−1^), remifentanil (0.2–0.3 μg·kg^−1^·min^−1^)	None
		50	Sevoflurane	Sevoflurane (1.0–1.5 MAC) remifentanil (0.2–0.3 μg·kg^−1^·min^−1^)	
Guo et al., 2020	Etomidate, sufentanil, rocuronium	117	Propofol	Propofol, sufentanil, rocuronium	None
		117	Sevoflurane	Sevoflurane, sufentanil, rocuronium	
Li et al., 2021	Fentanyl, lidocaine, propofol, and cisatracurium	226	Propofol	Intravenous propofol infusion (50 to 150 μg kg^−1^·min^−1^) and remifentanil infusion (0.1 to 0.5 μg · kg^−1^ · min^−1^).	No limitations for the use of muscle relaxant and vasoactive medications. Glucocorticoid drugs, nonsteroidal analgesics, and dexmedetomidine were avoided during surgery.
		221	Sevoflurane	Sevoflurane (1.0 to 1.5 MAC) and intravenous remifentanil infusion (0.1–0.5 μg kg^−1^ min^−1^)	
Liu et al., 2017	1.5 mg/kg propofol+2 μg/kg remifentanil+0.1 mg/kg vecuronium bromide intravenous (group P)	56	Propofol	3 ng/mL propofol and 4 ng/mL remifentanil	None
	2 mg/kg propofol+2 μg/kg remifentanil+0.1 mg/kg vecuronium bromide (group S)	56	Sevoflurane	2 mg/kg/h propofol+2 μg/kg/min remifentanil were continuously given through intravenous injection and vecuronium bromide was discontinuously given in the surgery to maintain muscle relaxation	
Mei et al., 2020	1–2 mg midazolam preoperatively, Propofol 2 mg/kg, sufentanil 0.5–1 μg/kg, cisatracurium 0.5 mg/kg,	106	Propofol	Propofol (629.8 ± 255.0 mg) by TCI	None
	Methylprednisolone (40–80 mg), atropine (0.25–1 mg)	103	Sevoflurane	Received 1–4% sevoflurane	
Nishikawa et al., 2004	Targeted propofol concentration of 4 mg/mL using a computer-assisted TCI system (group P)	25	Propofol	Vecuronium (1–2 mg i.v. boluses) +epidural analgesia with 1.5% lidocaine (4–6 mL/h)	The induction in both groups was combined with an epidural analgesia, 6–8 mL of 1.5% lidocaine solution, injected.
	5% sevoflurane and 100% oxygen at 6 L/min until the inspired-limb drug concentration was >4%.(group S)	25	Sevoflurane	Vecuronium (1–2 mg i.v. boluses) +epidural analgesia with 1.5% lidocaine (4–6 mL/h)	
Qiao et al., 2015	Intravenous injection of midazolam (2–3 mg), etomidate (0.3 mg/kg) and an infusion of sufentanil (0.4 μg/kg), cisatracurium besylate (0.3 mg/kg)	30	Propofol	Propofol administered by TCI (effect site concentration 4 μg/mL), intravenous infusion of remifentanil (commenced at 0.15 μg/kg/min and titrated according to clinical need) A 5 mg bolus of cisatracurium besylate was administered every 30 min according to clinical need.	None
		30	Sevoflurane	1 minimum alveolar concentration (MAC) sevoflurane, intravenous infusion of remifentanil (commenced at 0.15 μg/kg/min and titrated according to clinical need) A 5-mg bolus of cisatracurium besylate was administered every 30 min according to clinical need.	
Rohan et al., 2005	Target concentrations of propofol were adjusted to maintain adequate depth of anesthesia (group P)	15	Propofol	NR	After placement of an IV cannula and commencement of a 500 mL infusion of crystalloid solution in all patients, fentanyl 1 μg·kg^−1^ was administered intravenously.
	Incremental dose, tidal volume inhalation induction technique.(group S)	15	Sevoflurane	NR	
Tang et al., 2014	Propofol (1.5–2.0 mg/kg, i.v.) (group P)	101	Propofol	Propofol (6–10 mg/kg per h), remifentanil (9–12 mg/kg per h, continuous IV infusion), vecuronium (intermittent IV infusion)	Standard induction protocol: Midazolam (0.03–0.04 mg/kg, i.v.); fentanyl (0.002–0.003 mg/kg, i.v.); vencuronium (0.15–0.2 mg/kg, i.v.).
	8% sevoflurane (FGF 6 L/min, inhalation, decreased after loss of consciousness to 3–4%, FGF 1–2 L/min) (group S)	99	Sevoflurane	Sevoflurane (2–3%), remifentanil (9–12 mg/kg per h, continuous IV infusion), vecuronium (intermittent IV infusion)	
Zhang et al., 2018	Midazolam, remifentanil and/or sufentanil, propofol, and rocuronium or cisatracurium.	195	Propofol	Propofol infusion, remifentanil and/or sufentanil, rocuronium or cisatracurium.	None
		192	Sevoflurane	Inhaled sevoflurane remifentanil and/or sufentanil, rocuronium or cisatracurium.	

TCI, target-controlled infusion; MAC, minimum alveolar concentration; FGF, fresh gas flow; NR, no record.

Ten studies selected different methods of anesthesia maintenance. Geng et al. (20) used propofol (target concentration 2.5–3.0 μg·mL^−1^) and remifentanil (0.2–0.3 μg·kg^−1^·min^−1^) in group P, and sevoflurane (1.0–1.5 MAC) and remifentanil (0.2–0.3 μg·kg^−1^·min^−1^) in group S. Guo et al. (21) chose sufentanil and rocuronium with propofol or sevoflurane. Li et al. (3) used remifentanil (0.1–0.5 μg · kg^−1^ · min^−1^) with intravenous propofol infusion (50–150 μg · kg^−1^ ·min^−1^) or sevoflurane (1.0–1.5 MAC). Glucocorticoids, nonsteroidal analgesics, and dexmedetomidine were avoided during surgery. Mei et al. (18) used propofol (629.8 ± 255.0 mg) by TCI or 1–4% sevoflurane. Nishikawa et al. (27) chose anesthesia maintenance combined with continuous epidural analgesia with 1.5% lidocaine (4–6 mL/h). Qiao et al. (10) used an intravenous infusion of remifentanil (commenced at 0.15 μg/kg/min) and a 5-mg bolus of cisatracurium besylate was administered every 30 min according to clinical need with propofol administered by TCI (effect site concentration 4 μg/mL) or sevoflurane (1MAC). Tang et al. (23) chose remifentanil (9–12 mg/kg per h, continuous i.v. infusion) and vecuronium (intermittent i.v. infusion) with propofol (6–10 mg/kg per h) or sevoflurane (2–3%). Zhang et al. (24) used remifentanil (sufentanil) and rocuronium (cisatracurium) with propofol infusion or inhaled sevoflurane.

### Risk of bias assessment and study quality

**Figure 2:**
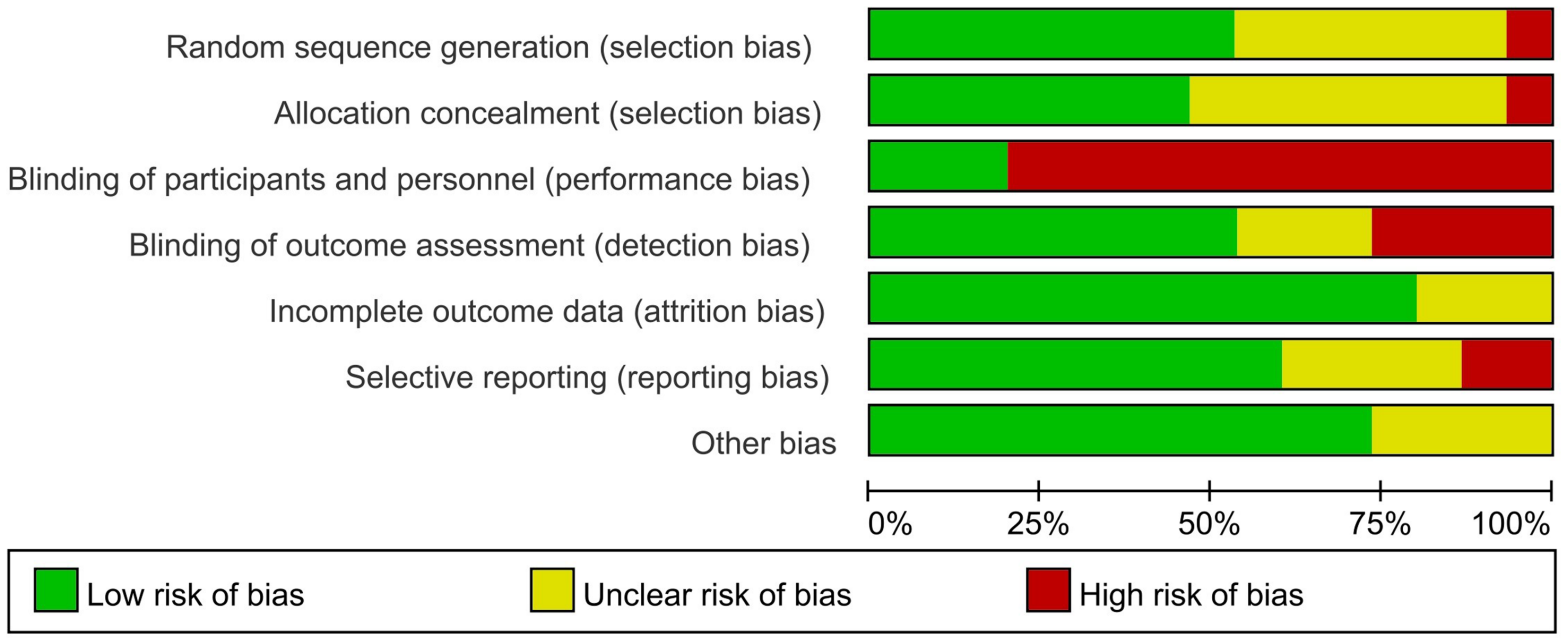
Risk of bias graph: review authors’ judgments about each risk of bias item presented as percentages across all included studies.

**Figure 3:**
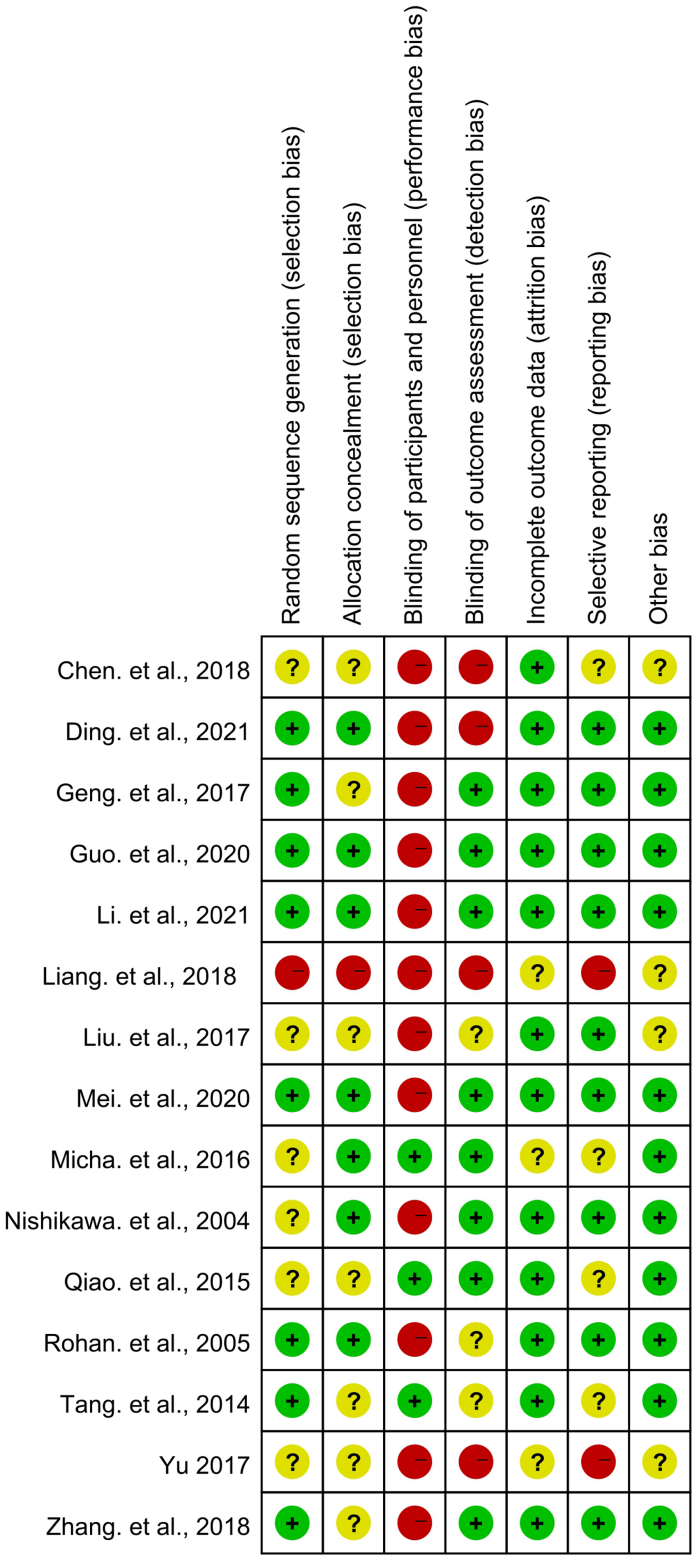
Risk of bias summary: review authors’ judgments about each risk of bias item for each included study.

We used RevMan 5.3 to summarize the bias of the included studies as shown in Figures 2, 3. It is denoted as high-risk, low-risk, or unclear. Of the 14 studies included in the risk of bias assessment, one (22) was a retrospective study with a high overall risk of bias. Eight studies mentioned random sequence generation methods, and seven described how to mask assignments. Six of the studies did not blind investigators and patients, and the outcome assessment was not blinded in four.

### Meta-analysis and synthesis

#### Incidence of POCD at 1, 3, and 7 days

**Figure 4:**
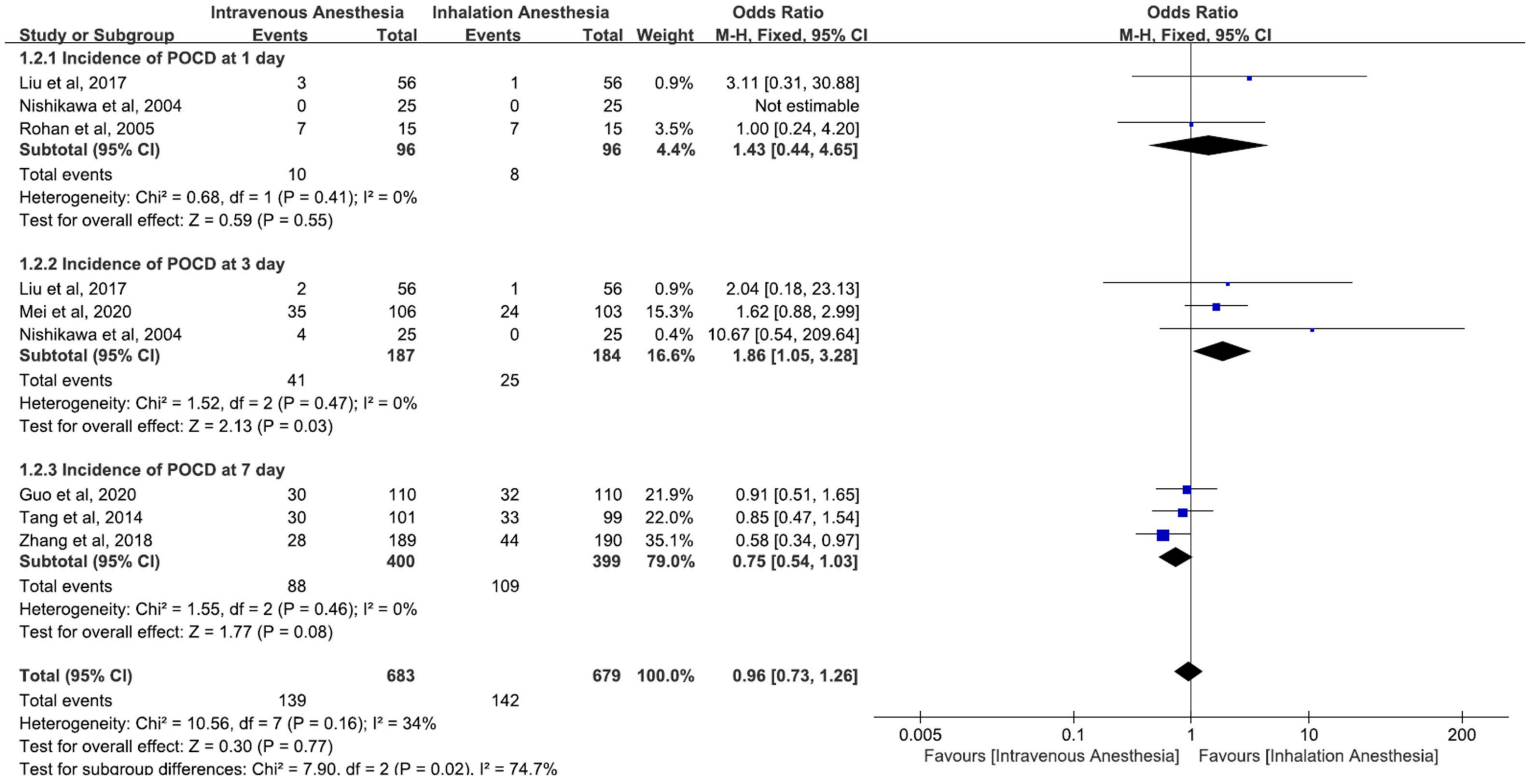
Incidence of POCD at 1–7 days postoperative comparison between intravenous anesthesia and inhalation anesthesia.

The incidence of POCD at 1 day was reported in a sample size of 192. Across three studies (17, 26, 27), the incidence of POCD at 1 day was 3, 0, and 7 in the intravenous anesthesia group. The incidence of POCD at 1 day was 1, 0, and 7 in the inhalation anesthesia group. The pooled OR (95% CI) of it was 1.43 (95% CI 0.44–4.65), I (2) = 0%, *n* = 3 (Figure 4). Using a fixed-effects model, the result was not statistically significant (*p* = 0.55).

Studies reported the incidence of POCD at 3 days with a sample size of 371. Across three studies (17, 18, 27), the incidence of POCD at 3 days was 2,35, and 4 in the intravenous anesthesia group. The incidence of POCD at 3 days was 1, 24, and 0 in the inhalation anesthesia group. The pooled OR (95% CI) of it was 1.86 (95% CI 1.05–3.28), I (2) = 0%, *n* = 3 (Figure 4). Using a fixed-effects model, the result was statistically significant (*p* = 0.03).

Studies reported the incidence of POCD at 7 days with a sample size of 821. Across three studies (21, 23, 24), the incidence of POCD at 7 days was 30, 30, and 28 in the intravenous anesthesia group. The incidence of POCD at 7 days was 32, 33, and 44 in the inhalation anesthesia group. The pooled OR (95% CI) of it was 0.75 (95% CI 0.54 to 1.03), I (2)= 0%, *n* = 3 (Figure 4). Using a fixed-effects model, the result is not statistically significant (*p* = 0.08).

Studies reported the incidence of POCD at 1, 3, and 7 days with a sample size of 1,222. The pooled OR (95% CI) of it was 0.96 (95% CI 0.73 to 1.26), I (2) = 34%, *n* = 7 (Figure 4). Using a fixed-effects model, the result was not statistically significant (*p* = 0.77). The subgroup differences were statistically significant (*p* = 0.02).

#### Plasma S-100β protein levels

**Figure 5:**
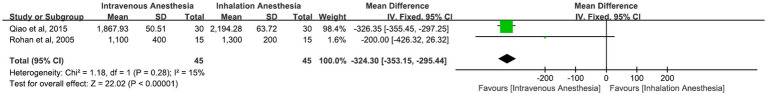
Plasma S-100β protein levels (pg*mL^−1^) postoperative comparison between intravenous anesthesia and inhalation anesthesia.

In two studies (10, 26), the plasma S-100β protein level (pg*mL^−1^) in the intravenous anesthesia group was 1867.93 ± 50.51 (Mean ± SD) and 1,100 ± 400 (Mean ± SD). The plasma S-100β protein level in the inhalation anesthesia group was 2194.28 ± 63.72 (Mean ± SD), and 1,300 ± 200 (Mean ± SD). The pooled MD (95% CI) of the plasma S-100β protein levels was −324.30 (95% CI −353.15 to −295.44) in favor of the intravenous anesthesia group, I (2)= 15%, *n* = 2 (Figure 5). Using a fixed-effects model, the result was statistically significant (*P <* 0.00001).

#### Delayed neurocognitive recovery incidence 5–7 days after surgery

**Figure 6:**
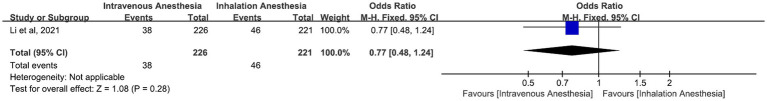
Delayed neurocognitive recovery incidence 5–7 days after surgery comparison between intravenous anesthesia and inhalation anesthesia.

In one study (3), the delayed neurocognitive recovery incidence 5–7 days after surgery in the intravenous anesthesia group was 38 and 46 in the inhalation anesthesia group. The pooled OR (95% CI) of it was 0.77 (95% CI 0.48–1.24), *n* = 1 (Figure 6). Using a fixed-effects model, the result was not statistically significant (*p* = 0.28).

## Discussion

We conducted a systematic review of 15 studies and a meta-analysis of 1,827 patients in 10 RCTs. Our results indicate that there was no significant difference between intravenous and inhaled anesthesia in the occurrence of POCD within 1–7 days and the incidence of delayed neurocognitive recovery 5–7 days after surgery. The plasma S-100β protein levels in the intravenous anesthesia group were lower than those in the inhalation anesthesia group.

The use of anesthetics has been a controversial topic and this review will provide an opinion on the choice of anesthetics for non-cardiac surgery in elderly patients. In our review and meta-analysis, sevoflurane was used to maintain anesthesia in the inhalation anesthesia group. We excluded desflurane and isoflurane considering that sevoflurane is preferred for induction or maintenance of anesthesia in most cases. At this point, our design differs from that of Miller et al. (29). Our results show that there was no significant difference between intravenous and inhalation anesthesia in the occurrence of POCD within 1–7 days after surgery. Moreover, the inhalation anesthesia group was better than the intravenous anesthesia group 3 days after surgery. This result was different from what we expected. This is because, according to previous studies (10, 20, 30, 31) and opinions, inhaled anesthetics will increase the risk of POCD in elderly patients. The reason for this difference may be related to the method of surgery, duration of anesthesia, and pre-operative medications. In a study favoring intravenous anesthesia, Qiao et al. (10) targeted elderly patients undergoing major surgery. The study by Geng et al. (20) was excluded from the sensitivity test due to significant heterogeneity. Furthermore, the conclusions of some recent studies can support our results. In a multicenter RCT designed by Li et al. (3), it was pointed out that the choice between propofol and sevoflurane did not affect the occurrence of POCD in elderly patients after laparoscopic surgery. Guo et al. (21) concluded in a double-blind RCT that sevoflurane did not significantly increase the incidence of POCD at 7 days and 3 months after surgery compared to propofol. In a study published in 2016 (32), sevoflurane use was associated with lower rates of POCD than propofol in patients with cerebral hypoxia. Recovery of neurocognitive function is strongly correlated with poor prognosis and the occurrence of adverse events after surgery. Before the study, Li et al. (3) assumed that the incidence of delayed recovery was lower in the propofol group than in the civilian group. They identified the incidence of delayed neurocognitive recovery 5–7 days after surgery as the primary outcome of the RCT. Controversy over the choice of anesthetic for non-cardiac surgery in elderly patients may persist in the context of unclear pathogenesis of POCD. However, without adequate research, it cannot be assumed that inhaled anesthetics are detrimental to a patient’s cognitive function.

S-100β protein has nerve tissue specificity and is a sensitive and specific marker of central nervous system damage (33, 34), especially in the elderly (35). Therefore, we chose the expression level of S-100β protein as an index to judge postoperative cognitive function. A meta-analysis conducted by Sun et al. (36) for postoperative cognitive function in elderly patients with lung cancer also added S-100β as an outcome indicator. Similar to our conclusion, Sun et al. also pointed out that the change in blood oxygen S-100β protein concentration in the sevoflurane group was significantly higher than that in the propofol group with statistical differences. These results all suggest that the use of pseudoephedrine to anesthetize older patients may result in more severe nerve damage and thus a greater risk of POCD than propofol. However, the measurement of S-100β as a laboratory indicator for assessing cognitive function may yield disparate results compared to behavioral studies, thereby accounting for the observed discrepancy in this study.

This meta-analysis has the following limitations. First, few articles were included in the systematic review because we had a rigorous literature screening, restricted the inhalation anesthesia group to intravenous anesthesia, and did not include studies published before 2000. In addition, we excluded studies with low quality and significant heterogeneity through a sensitivity analysis, so that the number of studies included in the meta-analysis was only 10. Second, this meta-analysis did not use the Mini-mental State Examination (MMSE) as an outcome indicator. In fact, we have performed subgroup analysis on the MMSE, but the heterogeneity of each subgroup was significant and the sensitivity analysis was limited. We believe that MMSE is a subjective approach. Also, the educational level, surgical method, and ASA classification of the patients included in each study were different, and most of the studies were from China. Finally, we did not analyze long-term outcomes because most POCD in the included studies occurred within 7 days of surgery. The long-term outcomes of non-cardiac surgical POCD in older patients have yet to be demonstrated.

## Conclusion

This systematic review and meta-analysis showed that for elderly patients undergoing non-cardiac surgery, inhalation anesthesia in general was comparable to intravenous anesthesia in terms of the occurrence of short-term POCD. Inhalation anesthesia may cause greater damage to the nervous system, with delayed recovery of cognitive function after 5–7 days showing no difference. Given the limitations of the included studies, we look forward to updating this review with more high-quality RCTs in the future.

## Data availability statement

The original contributions presented in the study are included in the article/supplementary material, further inquiries can be directed to the corresponding author.

## Author contributions

HL: Conceptualization, Data curation, Formal analysis, Investigation, Methodology, Software, Writing – original draft. ZY: Project administration, Resources, Writing – review & editing, Supervision, Validation.

## Abbreviations

PRISMA: Preferred Reporting Items for Systematic Reviews and Meta-analyses
BMI: Body Mass Index
SD: standard deviation
IQR: Inter Quartile Range
ASA: American Society of Anesthesiologists
TIVA: total intravenous anesthesia
MAC: minimum alveolar concentration
TCI: target-controlled infusion
POCD: postoperative cognitive dysfunction
FGF: fresh gas flow
NR: no record
MMSE: Mini-mental State Examination
